# Placental *Plasmodium falciparum *malaria infection: Operational accuracy of HRP2 rapid diagnostic tests in a malaria endemic setting

**DOI:** 10.1186/1475-2875-10-306

**Published:** 2011-10-18

**Authors:** Daniel J Kyabayinze, James K Tibenderana, Mercy Nassali, Lynette K Tumwine, Clare Riches, Mark Montague, Helen Counihan, Prudence Hamade, Jean-Pierre Van Geertruyden, Sylvia Meek

**Affiliations:** 1 Malaria Consortium, Upper Naguru East Road, P.O. Box 8045, Kampala, Uganda; 2 Unit International Health, ESOC Department, Faculty of Medicine, Antwerp University, Universiteiplein 1, BE-2610 Antwerpen, Belgium; 3 London School of Hygiene and Tropical Medicine, Keppel Street, London, WC1E 7HT, UK; 4 Mbale Regional Referral Hospital, Ministry of Health, PO Box 192, Mbale, Uganda; 5 Makerere University, College of Health Sciences, PO Box 7072, Kampala, Uganda; 6 Malaria Consortium, Development House, 56-64 Leonard Street, London, EC2A 4LT, UK

## Abstract

**Background:**

Malaria has a negative effect on the outcome of pregnancy. Pregnant women are at high risk of severe malaria and severe haemolytic anaemia, which contribute 60-70% of foetal and perinatal losses. Peripheral blood smear microscopy under-estimates sequestered placental infections, therefore malaria rapid diagnostic tests (RDTs) detecting histidine rich protein-2 antigen (HRP-2) in peripheral blood are a potential alternative.

**Methods:**

HRP-2 RDTs accuracy in detecting malaria in pregnancy (MIP >28 weeks gestation) and placental *Plasmodium falciparum *malaria (after childbirth) were conducted using Giemsa microscopy and placental histopathology respectively as the reference standard. The study was conducted in Mbale Hospital, using the midwives to perform and interpret the RDT results. Discordant results samples were spot checked using PCR techniques.

**Results:**

Among 433 febrile women tested, RDTs had a sensitivity of 96.8% (95% CI 92-98.8), specificity of 73.5% (95% CI 67.8-78.6), a positive predictive value (PPV) of 68.0% (95% CI 61.4-73.9), and negative predictive value (NPV) of 97.5% (95% CI 94.0-99.0) in detecting peripheral *P. falciparum *malaria during pregnancy. At delivery, in non-symptomatic women, RDTs had a 80.9% sensitivity (95% CI 57.4-93.7) and a 87.5% specificity (95%CI 80.9-92.1), PPV of 47.2% (95% CI 30.7-64.2) and NPV of 97.1% (95% CI 92.2-99.1) in detecting placental *P. falciparum *infections among 173 samples. At delivery, 41% of peripheral infections were detected by microscopy without concurrent placental infection. The combination of RDTs and microscopy improved the sensitivity to 90.5% and the specificity to 98.4% for detecting placental malaria infection (McNemar's *X *^2^> 3.84). RDTs were not superior to microscopy in detecting placental infection (McNemar's *X *^2^< 3.84). Presence of malaria in pregnancy and active placental malaria infection were 38% and 12% respectively. Placental infections were associated with poor pregnancy outcome [pre-term, still birth and low birth weight] (aOR = 37.9) and late pregnancy malaria infection (aOR = 20.9). Mosquito net use (aOR 2.1) and increasing parity (aOR 2.7) were associated with lower risk for malaria in pregnancy.

**Conclusion:**

Use of HRP-2 RDTs to detect malaria in pregnancy in symptomatic women was accurate when performed by midwives. A combination of RDTs and microscopy provided the best means of detecting placental malaria. RDTs were not superior to microscopy in detecting placental infection. With a high sensitivity and specificity, RDTs could be a useful tool for assessing malaria in pregnancy, with further (cost-) effectiveness studies.

## Background

*Plasmodium falciparum *malaria remains a major contributor of the disease burden in sub-Saharan Africa (SSA). Children under five years of age and pregnant women represent the most vulnerable population. During pregnancy, malaria infection leads to parasite sequestration in the maternal placental vascular space, with the consequence of increased risks of abortion, stillbirth, prematurity, intra-uterine growth retardation (IUGR) and maternal anaemia [1]. Malaria in pregnancy (MIP) is associated with increased risk of low birth weight (LBW) and prenatal, neonatal and infant mortality [2-5]. Infection with *P. falciparum *towards the end of gestation increases the likelihood of placental infection [6]. A previous review by Desai *et al *estimated that approximately one in every four pregnant women in malaria endemic areas has evidence of placental infection at the time of delivery [7].

The diagnostic challenge is that conventional peripheral blood microscopy is unable to detect all infections as parasites can be sequestered in the placenta [8-10]. Such sub-microscopic infections increase the risk of anaemia and poor foetal outcomes in seemingly aparasitaemic pregnant women [9,11] thus increasing the risk of maternal and perinatal mortality. Consequently, some clinicians are reluctant to withhold anti-malarials for febrile pregnant women, arguing that microscopy misses placental infections.

However, malaria rapid diagnostic tests (RDTs) may provide a solution as emerging evidence suggests that RDTs are capable of detecting placental malaria better than microscopy and may detect sub-microscopic infections [12,13]. RDTs are lateral flow immuno-chromatographic dipstick assays that detect either histidine rich protein-2 (HRP-2) or Plasmodium lactate dehydrogenase (pLDH) produced by infected red blood cells. Although the large majority of RDTs comprise mAbs raised against HRP2 or pLDH, it should be noted here that there are an increasing number of tests that also include a Pan anti-aldolase test line. Whereas good quality microscopy is lacking in many resource-limited settings, as it requires well-trained, competent personnel, infrastructure as well as effective quality control and quality assurance [13], RDTs are becoming increasingly available and affordable[14].

With many countries in SSA following World Health Organization (WHO) policy [15] and scaling up parasite-based diagnosis for malaria through the use of microscopy and RDTs, establishing the effectiveness of RDTs in comparison to microscopy in detecting malaria during pregnancy has become a priority.

Although there is limited evidence on the use of RDTs among pregnant women [16,17], RDTs have been found to be comparable in sensitivity to microscopy for malaria [18] and to improve malaria diagnosis and quality of care at lower level health facilities [19-21]. The few studies that have been done have based the accuracy of RDTs on either placental smear or placental blood polymerase chain reaction (PCR) and not histological examination of the placenta at delivery [10,11,22-24].

This study compares HRP-2 RDTs to microscopy for detecting peripheral malaria in feverish pregnant women at time of recruitment in a hospital setting. In addition, at delivery both RDTs and microscopy are compared to placental histopathology performed to detect placental malaria.

## Methods

### Study design and site

#### Study site

Mbale is a mountainous region with moderately low temperatures and humidity, approximately 300 km north east of Kampala, Uganda. It is a hyper-endemic region for malaria with an estimated parasite prevalence rate of 56% among children under five years [25]. The hospital conducts over 300 deliveries with less than 30% having attended antenatal care (ANC) in the same hospital. Insecticide-treated net (ITN) ownership in the region is 52% and net use by pregnant women is 42% [25]. Use of ANC services has been reported to be as high as 90%, and coverage with the second dose of intermittent preventive treatment (IPT) of malaria in pregnancy (IPTp-2) with sulphadoxine-pyrimethamine (SP) is below 50% [26]. The operational research was to determine the effectiveness of RDTs when used in a hospital setting. Cross-sectional evaluation at time of recruitment compared accuracy of RDTs to microscopy as a reference standard in detecting peripheral parasitaemia during pregnancy. At delivery, a second cross-sectional evaluation compared accuracy of RDTs and microscopy to the reference standard of placental histology performed to detect placental malaria.

#### Study participants

Patients with fevers (temperature >37.2) or history of fever in the last 24 hours, at a gestational age >28 weeks by dates, were screened and consented to participate in the study while at the antenatal clinic. Enrolled women were clinically examined and information was collected on social demographics and health-seeking behaviour using a pre-tested questionnaire. A code was placed on the ANC card of the participating mothers to enable easy identification and follow-up at time of labour. Pregnant women were encouraged to come and deliver in the hospital. All study participants were given transport refund as well as a free delivery set (maama kit) to ensure safe delivery. A finger prick was performed to test for malaria using both RDTs and microscopy smears. In addition, a sample of blood was collected on filter paper. Haemoglobin (Hb) testing was done using a portable spectrophotometer (HemoCue, Angelholm, Sweden). A routine (non-study) field stain smear, was performed to guide. Women with malaria, according to routine microscopy, were treated with either quinine or artemisinin combination therapy (ACT) according to the national treatment guideline.

During labour, participating mothers were identified based on a code placed on their ANC cards. A finger prick was performed to test for malaria using both RDTs and microscopy and to estimate Hb. A sample of blood was again collected on filter paper for PCR. After delivery, the outcomes and weight of the child were documented. A placental biopsy for histopathology was also performed. Mothers were requested to bring children back to the hospital for immunization and review after six weeks.

### Laboratory procedures

#### Rapid diagnostic testing for malaria

The commercially available RDT kit, "diagnosticks" Malaria *P. falciparum *cassette (SSA Diagnostics and Biotech Systems, Goa, India) was used. The lot number was: S21004, manufactured in September 2009 and expiry date of August 2011. Each box of 25 individually sealed strips with a desiccant was supplied with loops, alcohol swabs and lancets. Temperature and humidity conditions were monitored during transportation and storage. After performing a finger prick, two midwives independently read each of the RDTs, 15 minutes after adding five drops of the wash buffer according to the manufacturer's instructions. A result was interpreted as positive when both the test line and control line showed pink and the test line was also graded as either a strong or faint band. The result was recorded as negative when only the control line showed pink or invalid when the control line did not show up at all. Only one test was performed for each patient. No repeat test was done for invalid tests and used RDTs were discarded the same day. First reading was considered for analysis in the case of discordant result between readers.

#### Microscopy

Smears for the study were stained with 10% Giemsa for 30 minutes and examined under X100 objective lens of the microscope by two independent laboratory technologists blinded to each other's results. Microscopy examination of smears was done under oil immersion. The number of asexual parasites was counted against 200 leucocytes assuming an average leucocytes count of 8,000/µL. Before a smear was declared negative, 200 high power fields were examined.

#### Histopathology assessment of placental tissue

For every delivery conducted, placental biopsy was collected within 30 minutes to determine malaria infection. The specimen of the placental tissue (1.5 cm^3^) was excised away from the centre of the edge of the placenta through the full thickness of the placenta including the membranes [27]. Placental tissues were fixed in 10% neutral buffered formalin and transferred to the pathology laboratory at Makerere University, College of Health Sciences. At the pathology laboratory, the fixed placental biopsy specimens were grossed into 2-3 mm sections, processed using standard procedures and embedded in paraffin wax. They were sectioned using a microtometo 4µm [27]. The final tissue sections were stained with haematoxylin and eosin for detection of active malaria. Placental malaria infection was classified using the following definitions: 1) no infection (no evidence of parasites or pigment), 2) active acute infection (parasites in the maternal erythrocytes in the intervillous space but no/minimal pigment in fibrin/cells within fibrin), 3) active chronic infection (parasites in maternal erythrocytes in intervillous space, pigment in erythrocytes and circulating monocytes within intervillous space and pigment in fibrin or cells within fibrin and/or chronic villous syncytiotrophoblast/stroma and, 4) past infection (parasites not present, pigment confined to fibrin or cells within fibrin [28]. The enrolled sample of 434 pregnant febrile women at ANC was sufficient to detect a minimum sensitivity of 90% and specificity of 75% at a precision of 0.05 and power of 80%. The prevalence of malaria was estimated at 40% [28].

### Molecular assessments

A convenient sample of 50% of all discordant results (RDTs and microscopy) was selected for molecular assessment due to limited resources. Using the filter papers samples collected, PCR was performed to identify presence of infection in samples positive by microscopy, that had negative RDTs and samples negative by microscopy but positive by RDTs. Parasite DNA was isolated from filter paper using the chelex extraction method [29]. To detect *P. falciparum *, the block-3 region of merozoite surface protein-2 (MSP-2) was amplified by nested PCR with primers corresponding to conserved sequences flanking this region [30], followed by primers to amplify the *IC3D7 *and *FC27 *allelic family, using conditions described previously [31]. PCR products were analysed by electrophoresis using 2% agarose gel.

### Quality control measures

All study research assistants (midwives) were centrally trained and had continuous support supervision by the laboratory technician and site supervisor. The study RDTs were centrally purchased and delivered to the hospital by the study coordinator, with a reserve kept at the Malaria Consortium offices. Manufacturer's storage temperature specifications (4-30°C) were monitored and maintained both at storage and during transportation. The slides and RDTs were read by two persons blinded to each other's outcome. One data tracker was responsible for aggregating the data onto the case record forms. The practice of recording this information was checked during monthly support supervision visits. Placental histology smears were first examined by a senior pathologist and later, blindly, quality controlled by a second reader at Mbarara University pathology department. No tie-breaker reading was performed.

### Statistical methods

Clinical and social demographic data were collected at the time of enrolment and linked through unique identifier numbers. At delivery, outcomes of smear and placental histology results were also recorded in addition to repeat RDTs and peripheral microscopy. Data were entered using EpiData ("The EpiData Association" Odense, Denmark) and analysed with SPSS 12 (SPSS USA 2005). For malaria in pregnancy, results were categorized as positive and negative for both RDTs and microscopy. Sensitivity, specificity PPV and NPV were calculated by comparing the proportion of positive and negative results for each RDT with expert field microscopy (these are not certified WHO experts). Categorical variables were compared using chi ^2^test. Weighted positive likelihood ratio (LR) was calculated by dividing probability that a positive test result is a true positive by probability that a positive test result is a false positive as LR = (prevalence)(sensitivity)/(1-prevalence)(1-specificity) and the negative LR = (prevalence)(1-sensitivity)/(1-prevalence)(specificity). Logistic regression analysis was conducted to determine factors that predicted malaria test results and level of associations presented as odds ratios.

Placental results were categorized as positive (acute and chronic infections) or negative (past and not infected) for histopathology. Associations between placental infection and independent variables were assessed using odds ratios with 95% confidence intervals calculated and interpreted as significant if two-sided *p *-value was less than 0.05. For placental malaria, the sensitivity, specificity, PPV and NPV were calculated for both RDTs and microscopy in comparison to the reference standard placental histology. The McNemar test (X^2^) was used to examine whether the two *proportions *derived from the marginal sums of the matched sample contingency table significantly differed. McNemar's test result was computed: X^2 ^= ∑ (O-E)^2^/E where O is observed, E is expected value at 5% significant level [32]. Odds ratios and 95% confidence intervals of the discordant cells were computed to show the level of this association.

### Ethical considerations

The study was conducted according to the principles of the declaration of Helsinki and the international guidelines of biomedical research involving human subjects. The study protocol was approved by Mbale Hospital Research and Ethical Committee and Uganda National Council of Science and Technology. All participants provided written informed consent to participate in the study.

## Results

### Patient profile

**Figure 1:**
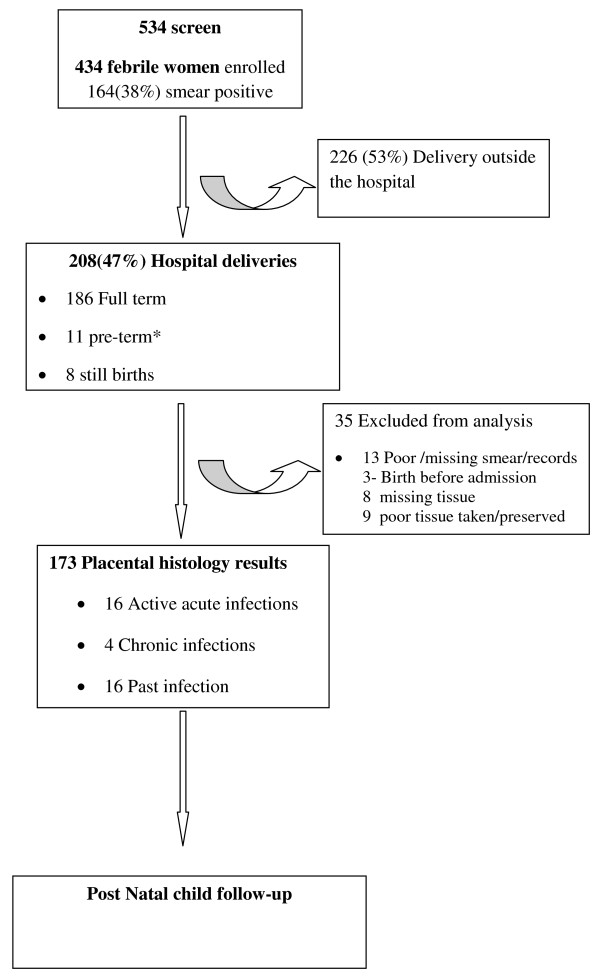
**Study profile of participants in the study to detect placental malaria infection using RDTs in Mbale Regional Hospital, Uganda**. The figure shows the study conduct when women were screened, enrolled and followed up to delivery and the samples included in the final analysis

Between February and July 2010, 534 febrile women attending ANC clinic were screened for eligibility to participate in the study, out of which 434 were enrolled. The screen failures were due to low gestational age (< 28 weeks of amenorrhoea). After testing with RDTs at time of enrolment, four women had invalid results and were excluded from analysis while one had no microscopy results. At delivery, 208/434 (48%) returned to the hospital. From the delivery data, 173/208 (83%) was analysed after excluding 28 without placental biopsies histology results and seven women who lacked unique linking data for both microscopy and RDTs results (Figure 1).

### Malaria in pregnancy (gestational malaria)

One third of the febrile women were primigravidae with a mean age of 19 years. Of these, 406/434 (94%) had attended ANC two or more times with just 58/434 (13%) having received a second dose of sulphadoxine-pyrimethamine for IPTp at time of enrolment. Among 421 respondents, 343 (82%) reported having slept under a mosquito net the night before. Prevalence of clinical malaria in pregnancy was 164/433 (38%) based on peripheral blood Giemsa smears microscopy, while RDTs were positive among 225/430 (52%)(Table 1). RDTs had a sensitivity of 96.8% (95% CI 92.0-98.8), specificity of 73.5% (95% CI 67.8-78.6), Positive Predictive Value (PPV) of 68.0% (95% CI 61.4-73.9), and Negative Predictive Value (NPV) of 97.5% (95% CI 94.0-99.0). The weighted likelihood ratios (LR) of positive test (LR+) and negative tests (LR-) were calculated. The LR+ was 2.1 (95% CI 1.7-2.6) which means that a pregnant woman with malaria is about two times more likely to have a positive RDT test than a woman who has not got malaria in pregnancy. A LR- of 0.025 (95% CI 0.01-0.06) means that the probability of having a negative test for pregnant women with malaria is 0.025 times that of those without malaria. Women without malaria are about 40 times more likely to have negative test than women with malaria. Given the LR, RDTs were better at 'ruling out malaria infection' than in confirming active malaria infection (Table 2). PCR was selectively conducted as a spot check to evaluate discordant results on a total of 45 'false RDTs' samples. PCR was positive for 1/37 RDT false negative and negative for 2/8 RDT false positive samples. These spot check PCR results did not affect the computed accuracy of the RDTs.

**Table 1:** Baseline characteristics of 434 febrile pregnant women enrolled in the malaria rapid diagnostic test in the antenatal clinic, January- Sept 2010 at Mbale Hospital, Uganda

Demographic characteristic	All women (N = 434)	Primiparae 131(30%)	Para II & III 153(36%)	Multiparae 150 (34%)
Educational ≤7 y(%,n)	52 (216/434)	41(51/123)	50 (71/151)	70(96/147)
Age yrs, (median, IQR range)	24(20-28)	19(15-37)	23(16-39)	30(17-42)
Teenager (%,n)	22 (146/434)	75(98/131)	26(40/153)	3(4/150)
WOA(mean, range)	32(28-36)	33(28-41)	32(35-40)	32(23-40)
Married %(n)	91(387/434)	77(101/131)	93(142/149)	96(144/150)
HIV Positive %(n)	5.5(24/434)	5.7(7/122)	5.3(8/151)	6.2(9/144)
Unknown HIV status%(n)	8.5(36/434)	9.0(11/122)	6.5(10/153)	10.4(15/144)
Anaemia % (n) Hb <11 gldl	43(185/150)	57 (74/131)	45(69/153)	39(59/150)
Hb <8 g/dl %(n)	4(17/434)	4.6 (5/131))	2(3/153)	5.3(8/150)

Back pain%(n)	19(82/434)	21(28/131)	22(33/153)	14(21/150)
Abdominal pain %(n)	24(103/434)	22(29/131)	21(32/153)	28(42/150)
Headache % (n)	19 (82/434)	38(50/131)	28(43/153)	36(54/150)
Temperature (°C, mean ±SD)	37.0 ± 0.9	37.2 ± 0.9	36.9 ± 0.8	36.9 ± 0.8
ANC visits ≥2 visits %(n)	94 (406/434)	94(124/130)	72(94/131)	93(137/147)
IPTp-1%(n)	62 (269)	69(75/108)	72(94/131)	78(100/127)
IPTp-2%(n)	13.4(58/434)	13(17/131)	15(23/153)	17(26/150)
Slept under net %(n)	82(343/434)	74(96/129)	83(123/148)	88(126/144)

DIAGNOSTIC TOOLS				

Routines smear positive(%,n)	49(205/434)	64(80/125)	53(77/147)	37.2(57/148)
HRP-2 positive(%,n)	52(225/430)	66.4(87/131)	53(81/152)	39.3(59/150)
Microscopy positive (%,n)	38(164/433)	51(67/131)	41(63/152)	23(34/150)

**Table 2:** Accuracy of peripheral blood RDTs in detecting malaria in pregnancy among 432 febrile women attending antenatal care at Mbale regional referral hospital between March and November 2010

Accuracy Parameter¶	Peripheral RDTs for symptomatic pregnant women(95% CI)
Sensitivity	96.8(92.3-98.8)
Specificity:	73.5(67.8-78.6)
Positive predictive value	68.0(61.4-73.9)
Negative predictive value	97.5(94.0-99.0)
**Likelihood ratio**	
Positive LHR[W]	2.13(1.72-2.62)
Negative LHR [w]	0.03(0.01-0.06)

Test sensitivity = conditional probability that the test will be positive if the condition is present

Test specificity = conditional probability that the test will be negative if the condition is absent

W = weighted for prevalence

### Factors associated with malaria in pregnancy

Primigravidae were more than twice as likely to have clinical malaria when compared with multigravidae women (aOR 2.5, p < 0.001). Women with low Hb were more at risk of having malaria than those with Hb greater than 11 g/dl (aOR 1.6, p = 0.024). Women who reported having slept under a mosquito net the night before were two times less likely to have malaria than those who did not use a mosquito net (aOR 0.44 p = 0.001) (Table 3).

**Table 3:** Frequency and risk factors of *Plasmodium falciparum *in 432 febrile pregnant women attending antennal clinic Mbale Hospital between February and October 2010

Parameter	Measure(n)	Malaria infection*	cOR	aOR	P Value
**Parity**	Primigravidae(n = 212)	105 (50%)	2.72 (1.82-4.07)	2.48 (1.64-3.75)	0.001‡
	≥2 pregnancies(n = 220)	59(27%)	1.0	1.0	
**Age**	<20 years (n = 141)	70(50%)	2.06(1.37-3.12)	1.09(0.63-1.92)	0.739
	≥20 years (291)	94(32%)	1.0	1.0	
**Maternal Anaemia**	<11.0 g/dL (n = 202)	91(45%)	1.76(1.19-2.61)	1.61(1.06-2.43)	0.024‡
	≥11.0 g/dL (n = 230)	73(32%)	1-0	1.0	
**HIV sero-status**	Positive or Unknown (n = 76)	34(44%)	1.41(0.85-2.32)	1.18(0.68-2.03)	0.563
	Negative (n = 356)	130(37%)	1.0	1.0	
**Gestational age (WOA)**	35 > weeks (n = 132)	59(45%)	1.50(0.98-2.27)	1.07(1.01-1.21)	0.018‡
	< 35 weeks (n = 300)	105(35%)	1.0	1.0	
**Previous IPTp use**	Yes (n = 296)	108(37%)	0.82(0.54-1.24)	1.23(0.78-1.92)	0.368
	No(n = 136)	56(41%)			
**Uses Net (ITN)**	Yes (n = 343)	115(34%)	0.41(0.26-0.66)	0.47(0.26-0.71)	0.001‡
	No (n = 89)	49(55%)	1.0	1.0	
**Education**	None or less than 7 years(n = 228)	89(39%)	0.91(0.62-1.34)	1.25(0.82-1.90)	0.298
	More than 7 years(n = 204)	75(37%)	1.0	1.0	

*Peripheral smear positive

### Placental malaria and its associated factors

At the end of gestation, 208/434 (48%) enrolled mothers returned for hospital delivery. The delivery outcomes were 186/208 (89%) live births, 11 pre-term, eight still births (five fresh still births), and three unrecorded outcomes. Six (3%) were surgical deliveries. One maternal death occurred due to post-partum haemorrhage and three neonatal deaths on the first-day of life due to prematurity. Further analysis was then restricted to 173 patients with complete histopathology results.

Of the 178 placental tissue examined, 38/178 (21.3% [95% CI 16.0-27.9]) had malaria infection, of which 21/173(12.1% [95% CI 7.8-17.4]) were active infections (16 acute and five chronic) and the rest past infections. RDTs had a sensitivity of 80.9% (95% CI 57.4-93.7), specificity of 87.5% (95% CI 80.9-92.1), PPV of 47.2% (95% CI 30.7-64.2) and NPV of 97.1% (95% CI 92.2-99.1) against the reference standard of placental histology in detecting placental *P. falciparum *infections. Combination results of RDTs and microscopy improved the total sensitivity to 90.5 (95% CI 68.2-98.3) for detecting placental malaria infection and NPV to 98.4 (95% CI 93.9-99.7) but lowered the specificity and PPV (Table 4). The McNemar's test (*X *^2^) was performed to test whether RDTs were better than microscopy in detecting placental malaria. The *X *^2 ^values among the placental-infected and non-infected mothers were 0.25 and 1.565 respectively, both less than the 3.84 cut-off for a significant difference at 5% (Additional file 1). The extended McNemar *X *^2 ^confirmed that combined tests of RDTs and microscopy were significantly better than a single test (*X *^2 ^of 6.125 for infected and 9.00 none infected both >than 5.00 cut off). At delivery, the mean age was 25 (SD 6.0) years and 47/166 (28%) were teenagers. The mean haemoglobin concentration was 11 (SD 3.0) g/dL. Overall, 64/161(40%) of the mothers were anaemic at delivery (Hb <11.g/dL). Using a logistic regression model, the clinical factors associated with placental infection were late pregnancy malaria infection (aOR = 20.9, p < 0.001) and poor foetal outcome (aOR = 37.9, p = 0.007). There was no statistically significant association between placental infection and ITN use, IPTp use and birth weight (Table 5).

**Table 4:** Accuracy of microscopy and RDT in detecting placental malaria infection in peripheral blood among 173 women at Mbale regional referral hospital between March and November 2010

		Placental histology				
		+(n = 21)	(n = 152)	Total	Accuracy measure(95%CI)	
Microscopy	+	**17**	**12**	29	Sensitivity	76.2(52.4-90.8)
		**4**	**140**	144	Specificity	92.1(86.3-95.7)
		
					PPV*	57.1(37.4-74.9)
					NPV‡	96.6(91.7-98.7)

RDTs	+	**17**	**19**	36	Sensitivity	80.9(57.4-93.7)
		**4**	**133**	137	Specificity	87.5(80.9-92.1)
		
					PPV	47.2(30.7-64.2)
					NPV	97.1(92.2-99.1)

Microscopy	+	**19**	**27**	46	Sensitivity	90.5(68.2-98.3)
+RDTs		**2**	**125**	127	Specificity	82.2(75.0-87.7)
		
					PPV	41.3(27.3-56.7)
					NPV	98.4(93.9-99.7)

**Table 5:** Frequency and risk factors of placental *Plasmodium falciparum *infection among 166 delivering mothers in Mbale Hospital between February and October 2010

Parameter	Categories(n)	Placental Infection	cOR	aOR	P Value
**Parity**	Primigravidae(n = 83)	14 (17%)	2.60 (0.95-7.15)	1.01 (0.21-4.72)	0.994
	≥2 pregnancies(n = 83)	6(7%)	1.0	1.0	
**Age**	< 20 years (n = 47)	9(19%)	2.33 (0.90-6.05)	1.68 (0.44-6.46)	0.451
	≥20 years (119)	11(9%)	1.0	1.0	
**Maternal Anaemia**	<11.0 g/dL (64)	11(17%)	2.31 (0.87-6.10)	2.09 (0.63-6.96)	0.222
	≥11.0 g/dL (n = 97)	8(8%)	1.0	1.0	
**Pregnancy outcome**	Still birth (n = 14)	6(36%)	1.34(0.16-11.12)	37.9 (2.70-528.80)	0.007‡
	Live full term(n = 149)	14(9%)			
**Birth weight**	< 2500 gm (n = 11)	1(9%)	1.34 (0.16-11.12)	17.04 (0.63-460.7)	0.092
	≥2500 gm(n = 97)	8(8%)			
**Previous IPTp use**	Yes (n = 118)	17(14%)	0.40 (0.11-1.42)	0.31 (0.07-1.40)	0.127
	No(n = 30)	7(23%)	1		
**Uses Net (ITN)**	Yes (n = 136)	13(10%)	0.34 (0.13-0.96)	1.02 (0.22- 4.58)	0.977
	No (n = 30)	7(23%)	1.0	1.0	
MIP*	Smear Positive(n = 61)	16(26%)	8.98 (2.84-28.37)	20.95 (3.99-110.05)	0.001‡
	Smear Negative(n = 105)	4(4%)	1.0	1.0	

*Malaria in pregnancy

aOR = Adjusted odds ratios

‡ Statistically significant

## Discussion

This study found a malaria prevalence of 38% using microscopy and 54% with RDTs among febrile pregnant women at ANC. When compared with microscopy, RDTs demonstrated acceptable sensitivity (97%) and specificity (74%) for the diagnosis of *P. falciparum *malaria among febrile pregnant women attending ANC at a hyper-endemic region in eastern Uganda. The procedures in this study were performed by nurses and midwives at the hospital ANC clinic. The operational nature of this evaluation allowed the investigators to demonstrate that RDTs are reliable for diagnosis of *P. falciparum *malaria in pregnancy when performed by midwives. These findings are comparable to previous reports of diagnosed malaria in pregnancy ranging from 9% to 60% in sub-Saharan Africa [33].

The high sensitivity of RDTs among symptomatic pregnant mothers is comparable to results from a previous operational study among outpatients in a similar setting [34]. The high sensitivity gives confidence to clinicians that RDTs are unlikely to miss malaria infections in pregnancy. The PPV was low and this could be due to false positive results attributable to the persistent nature of HRP-2 antigenemia already documented by previous studies [34-36]. The false positive may also have been due to better detection of malaria by HRP2 test in respect to the microscopy used as reference [37]. Using PCR to validate the false positives did not alter this result [37]. This was unexpected as the sensitivity of PCR has been documented as significantly higher than RDTs [17].

This study found a relatively low specificity but a high negative predictive value of 97.5%, almost certainly excluding malaria infection. This NPV result gives confidence that if RDTs are used to diagnose malaria in pregnancy very few infected women will be missed. Moreover, false negatives were linked to very low parasite densities that are unlikely to be the cause of illness in such an endemic setting. The consequences of a false negative in an endemic area, particularly when related to low parasitaemia, mean that the patient is unlikely to die. The infection may either clear without illness, as people living in endemic areas have partial immunity, or clinical symptoms will recur and they would seek care again.

For clinical application, sensitivity and specificity of the RDTs can be combined into a single measure called likelihood ratio (LR). LR provides a summary of how many times more (or less) likely patients with malaria are to have a positive (or negative) result than women without malaria. This study found a LR+ of two which means that a pregnant woman with malaria is about two times more likely to have a positive RDT test than a woman who has not got malaria in pregnancy. It also found a LR- of 0.025 which means that the probability of having a negative test for pregnant women with malaria is 0.025 times of that of those without malaria. Therefore, women without malaria are about 40 times more likely to have negative test than women with malaria. These results imply that RDTs were better at 'ruling out malaria infection' than in confirming active malaria infection. The benefit of likelihood ratios is that they can be used to help the clinician adapt the sensitivity and specificity to tests of individual patients [38,39]. In summary, the high sensitivity, specificity, NPV and the LR of RDTs in symptomatic pregnant women, demonstrated in this study, gives confidence to the midwife (or physician) to treat all RDT-positive and not treat women with a negative RDT. However, to make sure that all pregnant women receive the doses of SP for IPT that will hopefully clear any infection and placental infection if present, the midwife should investigate for non-malarial causes of fever when the RDT is negative.

This study found that there was an association between RDT result and clinical or demographic factors. During routine care, the clinician takes a history and examines the patient to estimate the chance (probability) of malaria prior to performing a test. A patient's probability of having malaria after the test result is known (post-test probability) is what the clinicians are most interested in as it can help in deciding where to confirm a diagnosis or rule it out. The actions taken after receiving the test results are weighed in light of the history and examination obtained prior to testing. The factors that influenced post-test probability in this study are consistent with known risk factors associated with malaria infection, and include parity, low maternal haemoglobin, sleeping under a mosquito net and advanced gestational age. Previous studies have shown that primigravidae are at increased risk for malaria in pregnancy and most cases of malaria in pregnancy happen towards the end of gestation [1,6,40,41]. The findings affirm the notion that HRP2 RDTs are most useful when the clinical and social demographic context is known because of varying risk levels for malaria during pregnancy [42].

In this study, direct examination of the placental tissue showed that 21% of women had placental infection of which 12% were active infections on the day of delivery. The study demonstrated a modest level of accuracy (80.9% sensitivity, 87.5% specificity, 98% NPV) of RDTs in detecting placental malaria using peripheral blood at time of delivery. The study allowed the investigators to characterize the burden of placental infection among pregnant women with history of febrile illness during the third trimester. The prevalence of placental malaria is similar to that reported from Nigeria [43]. However active placental malaria infection at the time of delivery in this study was lower than the reports from Cameroon at 60% [9] and 34% in a study in Ghana [11]. The possible reason for the difference with previous studies is that those studies used placental microscopy smears rather than histology to detect placental infection. In addition, it is possible that this study presents lower rates of placental malaria because of the high use of IPT-1 at 70% and ITN use at 84%. Use of IPT and ITNs reduces the risk of malaria and placental malaria [44]. The current study highlighted that as parity and use of ITNs increase, there is a significant reduction in the risk of placental malaria infection. There was no significant difference between RDTs and microscopy in detecting placental malaria. The combined sensitivity and specificity of both peripheral microscopy and RDTs detecting placental malaria was higher than using individual tests and this difference was statistically significant [32]. This finding is similar to that reported in a study in Cameroon where a combination of microscopy and RDTs detected more placental infection [10]. This implies that RDTs could complement peripheral microscopy in excluding placental malaria. The clinical relevance of this is that women with negative tests for both microscopy and RDTs should not be treated with anti-malarial drugs as chances of having placental malaria are minimal. However, intermittent presumptive treatment needs to be advocated for and promoted to minimize the risks of placental infection and the associated complications to the mother and foetus [3,11,45]. Malaria in pregnancy and acute placental infection increase the risk of clinical malaria in the mother, severe anaemia, intra uterine growth retardation and death in infancy [1]. In RDT negative pregnant women, IPT with SP should be given. Although some studies have reported good efficacy of SP, its future usefulness is questionable [46] given the current prevalence of mutant parasites [47,48], and yet alternative effective and safe replacement remains unclear. The sensitivity of the RDT in this study was acceptably high and any clinician who does not treat the patient with a positive RDT would be negligent, but should also explore other reasons for fever, which could in any case coexist with malaria. Some mothers had false positive RDTs without placental or peripheral infection detected. The consequence of false positive result is that someone may be treated for malaria when they are not infected. Based on these study results, health workers will over-treat fewer mothers than if they treated all symptomatic women. It is also likely that some of these malaria infections were identified early before migration of infection to the placenta as shown by positive peripheral smear readings [43].

This study was integrated into the existing health system and done by the human resources who would be available for eventual scale-up and in close collaboration with National Malaria Control Programme. The study built on previous work published by others by using an implementation/prospective approach and providing an appropriate context in which malaria is diagnosed. This approach in study conduct was important to validate effectiveness of RDTs given this can be context specific [42]. It has been argued that the effectiveness of RDTs is dependent on malaria transmission intensity and the operational context.

The main limitations of this study included the low hospital delivery rate of 47%, which made it impossible to analyse the deliveries out of the hospital. However the hospital delivery rate was higher than the national rate of 30% and the authors attribute this to the study mothers being incentivized to deliver in the hospital. Some samples were not analysed due to missing data, an expected challenge when using data from a routine ANC care setting. These limitations are to be expected in most operational studies but this approach allows the team to learn through implementation. Another limitation is that the RDTs (Diagnosticks) selected for the study displayed a very low sensitivity (59%) against low parasitaemia blood samples in an independent evaluation conducted by FIND/WHO [49]. Additionally, it has been noted that this RDT also has quite a high false positivity rate of 8% (8 out of 100 negative blood samples tested positive for *P. falciparum *). The inherently higher rate of false positives recorded for this test may explain the poor PPV observed in the study and also account for lower specificity percentages. It is recommended by the WHO that an RDT should have at least 95% sensitivity at 100 p/µl [50]. It is possible that another HRP-2 RDT would perform better in terms of sensitivity in this context. In order to provide a more comprehensive and thorough assessment of performance of an RDT when compared to microscopy or any other experimental method, future studies should make use of the WHO interactive online guide for selection of RDTs. Studies should include well characterized RDTs with much higher sensitivities and specificities and more than one test evaluated to provide a more robust assessment of their performance in this context.

## Conclusions

Prevalence of active placental malaria infection was 12% in a malaria endemic setting in Uganda. RDTs were accurate and comparable to microscopy for diagnosing malaria in pregnancy when performed by midwives during ANC. RDTs were not statistically better than microscopy in detecting placental infection and both methods were less than optimal. Combined use of RDTs and microscopy on peripheral blood improved detection of placental malaria infection. RDTs high sensitivity and specificity of RDTs means they can complement microscopy for diagnosing *P. falciparum *malaria in pregnancy with further (cost-) effectiveness studies.

## Competing interests

The author declares that they have no competing interests.

## Authors' contributions

DJK participated in the conception, study design, protocol development, training, and was responsible for project management, data analysis and interpretation, and developed the draft manuscript. NM, CR, DN, and MM participated in the development of tools, interpretation of results and writing of the manuscript. LKT and MN participated in the training of health workers and acquisition of data. HC, JP, SM and JKT participated in the conception study, interpretation of results, provided technical over sight and leadership in writing of the manuscript. All authors read and approved the final manuscript.

## Supplementary Material

Additional file 1 **Matched samples table for infected (n = 21) and non malaria infected placental (n = 152) readings comparing RDTs to microscopy reading based on histopathology as a reference standard among delivering mothers in Mbale Hospital between February and October 2010**. The table show the contingency table marginal totals used to compute the ***McNemar X *^2 ^**test comparing RDTs to microscopy reading based on histopathology as a reference standard among 173 delivering mothers in Mbale Hospital between February and October 2010. The *X *^2 ^values among the placental-infected and non-infected mothers were 0.25 and 1.565 respectively, both less than the 3.84 cut-off for a significant difference at 5%. The comparison in c and d compares the combined results of both RDTs and microscopy to that of RDTs alone (two degrees of freedom). The extended McNemar *X *^2 ^confirmed that combined tests of RDTs and microscopy were significantly better than a single test (*X *^2 ^of 6.125 for infected and 9.00 none infected both >than 5.00 cut off). Click here for file
