# Supplementary material for: Placental Plasmodium falciparum malaria infection: Operational accuracy of HRP2 rapid diagnostic tests in a malaria endemic setting
Source: Malar J. 2011 Oct 18;10:306. doi: 10.1186/1475-2875-10-306 (PMC3206496; doi:10.1186/1475-2875-10-306)
Supplement: Additional file 1 — Matched samples table for infected (n = 21) and non malaria infected placental (n = 152) readings comparing RDTs to microscopy reading based on histopathology as a reference standard among delivering mothers in Mbale Hospital between February and October 2010. The table show the contingency table marginal totals used to compute the McNemar X 2 test comparing RDTs to microscopy reading based on histopathology as a reference standard among 173 delivering mothers in Mbale Hospital between February and October 2010. The X 2 values among the placental-infected and non-infected mothers were 0.25 and 1.565 respectively, both less than the 3.84 cut-off for a significant difference at 5%. The comparison in c and d compares the combined results of both RDTs and microscopy to that of RDTs alone (two degrees of freedom). The extended McNemar X 2 confirmed that combined tests of RDTs and microscopy were significantly better than a single test (X 2 of 6.125 for infected and 9.00 none infected both >than 5.00 cut off). [file 1475-2875-10-306-S1.DOC]

## Additional Table: Matched samples for infected (n=21) and non malaria infected placental (n=152) readings comparing RDTs to microscopy reading based on histopathology as a reference standard among delivering mothers in Mbale Hospital between February and October 2010

| a) Matched samples for 21 malaria placental infected women comparing RDTs to microscopy according to histopathology | | | | | | | |
| --- | --- | --- | --- | --- | --- | --- | --- |
|  |  | RDTs | | |  | **Comparison** | ***McNemar X*^2^** |
|  |  | **+** | **­** | |  |  |  |
| Microscopy | + | 15 | **2** | | 17 | Infected | 1.375 |
|  | ­ | **2** | 2 | | 4 | OR discordant cells(95% CI) | 1.0(0.14-7.0) |
|  | Total | 17 | 4 | | 21 |  |  |
| b) Matched samples for 152 non malaria placental infected women according to histopathology | | | | | | | |
|  |  | **RDTs** | | |  | **Comparison** | ***McNemar X*^2^** |
| Microscopy | + | 4 | **8** | | 12 | Non infected | 0.210 |
|  | ­ | **15** | 125 | | 140 | OR discordant cells (95% CI) | 1.87(0.79-4.42) |
|  | Total | 19 | 133 | | 152 |  |  |
| c) Combined RDTs and microscopy matched samples for 21 malaria placental infected women according to histopathology | | | | | | | |
|  |  | **RDTs** | | |  | **Comparison** | ***McNemar X*^2^** |
| Microscopy +RDTs | + | 17 | **2** | | 19 |  |  |
|  | ­ | **0** | 2 | | 2 | Infected | 0.50 |
|  | Total | 17 | 4 | | 21 |  |  |
| d) Combined RDTs and microscopy matched samples table for 152 non malaria placental infected women according to histopathology | | | | | | | |
|  |  | **RDTs** | | |  | **Comparison** | ***McNemar X*^2^** |
| Microscopy +RDTs | + | 19 | | **8** | 27 | Non infected | 6.125* |
|  | ­ | **0** | | 125 | 125 | Combined test | 9.0‡ |
|  | Total | 19 | | 133 | 152 |  |  |

*Statistically significant for McNemar > 3.841 at 5% significant level.

‡ Statistically significant McNemar >5.99 for 2 degrees of freedom
